# *De novo* transcriptomic profiling of the clonal *Leymus chinensis* response to long-term overgrazing-induced memory

**DOI:** 10.1038/s41598-018-35605-y

**Published:** 2018-12-17

**Authors:** Weibo Ren, Xiangyang Hou, Zinian Wu, Lingqi Kong, Huiqin Guo, Ningning Hu, Dongli Wan, Jize Zhang

**Affiliations:** 1 Institute of Grassland Research, Chinese Academy of Agricultural Sciences, Hohhot, 010010 Inner Mongolia China; 2 College of Life Sciences, Inner Mongolia Agricultural University, Hohhot, 010019 Inner Mongolia China; 3 Institute of Genetics and Developmental Biology, Chinese Academy of Sciences, Beijing, 100101 China

## Abstract

Sheepgrass (*Leymus chinensis*) is one of the dominant grass species present on typical steppes of the Inner Mongolia Plateau. However, *L*. *chinensis* has developed a dwarfing phenotype in response to the stressful habitat in grasslands that are severely degraded due to heavy grazing. The lack of transcriptomic and genomic information has prohibited the understanding of the transgenerational effect on physiological alterations in clonal *L*. *chinensis* at the molecular level in response to livestock grazing. To solve this problem, transcriptomic information from the leaves of clonal *L*. *chinensis* obtained from overgrazed (GR) and non-grazed (NG) grasslands was studied using a paired-end Illumina HiSeq 2500 sequencing platform. First, despite the influence of grazing being absent during the growth of clonal offspring in our hydroponic experiment, compared with those from the NG group, clonal *L*. *chinensis* from the GR group exhibited significant dwarf-type morphological traits. A total of 116,356 unigenes were subsequently generated and assembled *de novo*, of which 55,541 could be annotated to homologous matches in the NCBI non-redundant (Nr), Swiss-Prot, Clusters of Orthologous Groups (COG), gene ontology (GO), or Kyoto Encyclopedia of Genes and Genomes (KEGG) databases. The expression of 3,341 unigenes significantly differed between the GR group and the NG group with an absolute value of Log_2_ ratio ≥ 1. The altered expression of genes involved in defence and immune responses, pathogenic resistance and cell development indicates that livestock grazing induces a transgenerational effect on the growth inhibition of clonal *L*. *chinensis*. The results of the present study will provide important large-scale transcriptomic information on *L*. *chinensis*. Furthermore, the results facilitated our investigation of grazing-induced transgenerational effects on both the morphological and physiological characteristics of *L*. *chinensis* at the molecular levels.

## Introduction

The grassland on the Inner Mongolian steppe is the most important region for the production of forage, mutton, and milk in China; the region covers 68% of the total land area of the Inner Mongolia Autonomous Region^1,2^. However, almost half of this region suffers from deterioration and desertification^3^. The major factor causing these problems is overgrazing, which threatens the sustainable development of grassland ecosystems^2–4^. Both the quality of forage and animal products decrease due to the effect of overgrazing^5^.

Grazing can lead to plant morphological, physiological and phenological adaptations, such as reduced shoot internodes or altered nutrient use rates; in turn, these adaptations can alter plant growth performance under various environmental stresses^6^. In response to animal grazing, steppe plants tend to exhibit resistance capabilities that affect growth performance, life history and biomass allocation^7–10^. These adaptations not only are a result of genotype × environment interactions but also can be passed down to progeny (referred to as transgenerational effects or stress-induced memory). Recent studies on transgenerational effects have experimentally demonstrated that local phenotypic adaptations under multiple stresses can be heritable in asexually produced offspring of clonal plants under both similar and contrasting environmental conditions^11,12^.

Natural steppe plants are continually confronted with various biotic and abiotic stresses due to seasonal alterations of their habitat^13^. A growing body of experimental evidence suggests that clonal steppe plants exhibit transgenerational effects when the maternal plant is exposed to such biotic and abiotic stresses^14^. For example, the results of a study in which seeds were collected from 58 grassland sites in Europe showed that the clonal herb *Trifolium repens* displayed strong vertical foraging via petiole elongation when grown either directly among or in close proximity to competitors, as the productivity of the maternal herb differed under different mowing and grazing intensities^15^. In addition, transgenerational effects of nitrogen and phosphorus enrichment on *Stipa krylovii* and *Artemisia frigida* have been reported in an Inner Mongolia grassland ecosystem, where clonal offspring of the two species exhibited different adaptive strategies to nutrient additions^16^.

Sheepgrass (*Leymus chinensis*) is one of the dominant species present in the typical steppe grasslands on the Inner Mongolian Plateau. Among the species in the *Leymus* genus, *L. chinensis* is an important forage species that is preferred by large herbivores; the species presents good quality, high palatability and nutrition value, and resistance to various stresses^17–19^. In recent decades, the degradation of *L*. *chinensis* on steppes has become increasingly obvious because of the presence of dwarf characteristics of the offspring due to overgrazing^20^. Understanding the mechanisms contributing to transgenerational effects on steppe plants in response to livestock grazing can provide insight into the biological processes of grazing-induced alterations in grassland ecosystem function. Our previous research on clonal transgenerational effects on grassland plants indicated that significant differences in leaf photosynthesis in *L*. *chinensis* subjected to long-term overgrazing or non-grazing were maintained in the clonal offspring in a greenhouse experiment designed to remove the maternal environment^21^. However, in addition to photosynthesis, information on the link between transgenerational effects and clonal plant physiological alterations at the molecular level in response to livestock grazing is scarce.

The development of next-generation sequencing (NGS) technology has been rapidly increasing, providing a large amount of sequencing information with the added advantage of increased efficacy and low cost^22,23^. As such, the objective of this study was to use an Illumina HiSeq 2500 sequencing platform to understand the relative transcriptional changes in the leaf transcriptome of clonal *L*. *chinensis* plants in which the maternal plant was grown under conditions of either long-term grazing or non-grazing.

## Results

### Illumina sequencing and reads assembly

**Figure 1:**
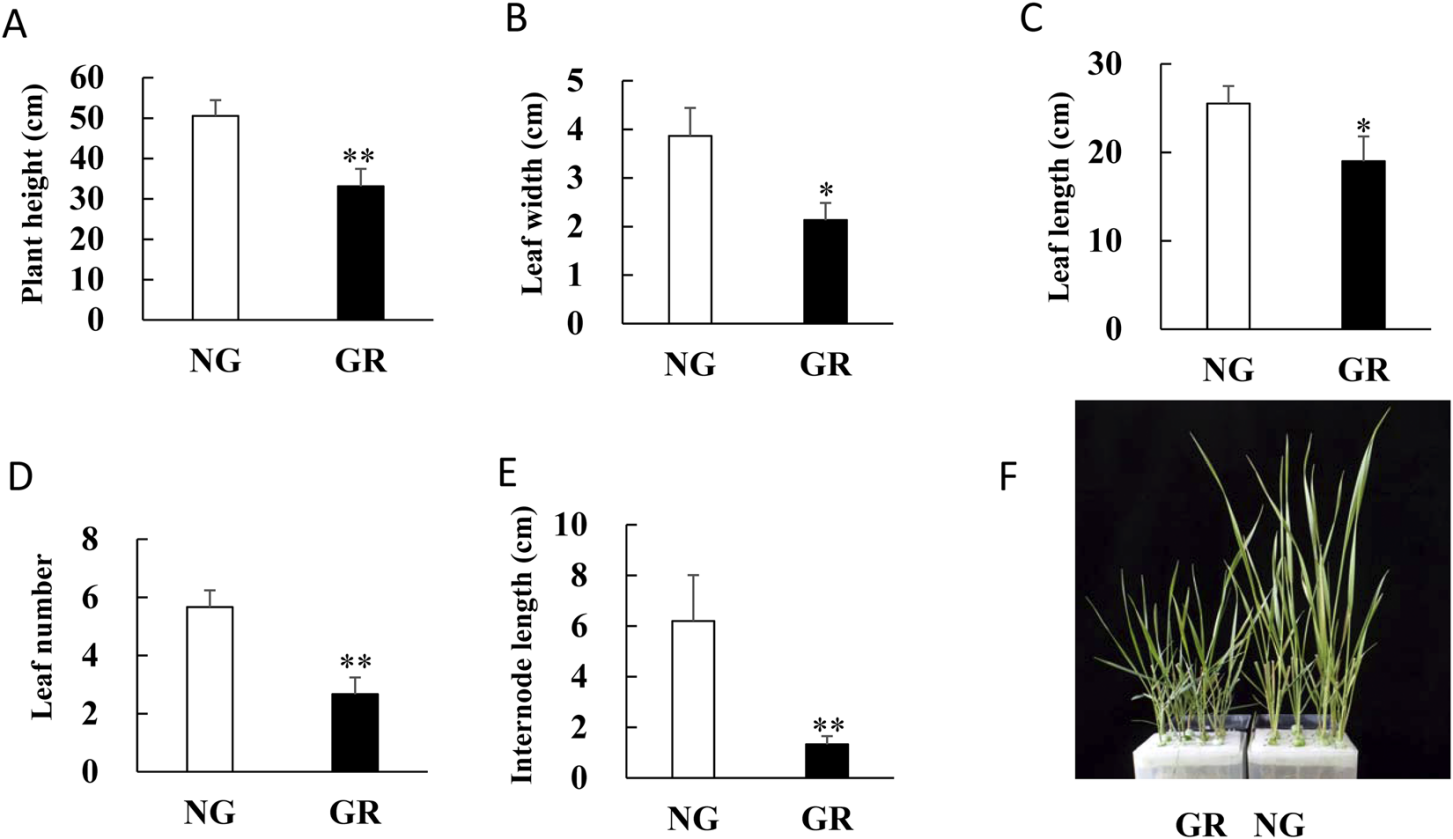
Differences in plant growth and multiple morphological traits in clonal *L. chinensis* in response to maternal plants grazing in a hydroponic experiment. (**A**–**E**) The morphological traits are indicated on the x-axis. (**F**) Photographs of clonal *L. chinensis* cultivated under hydroponic conditions. The cultured *L. chinensis* buds were collected from the GR and NG plots. GR, overgrazed; NG, non-grazed; Symbols: ^**^P < 0.01, *P < 0.05.

To investigate the different transcriptomic profiles between clonal overgrazed (GR) and non-grazed (NG) *L*. *chinensis*, the leaves of hydroponically grown clonal *L*. *chinensis* plants from both NG and GR areas were sampled. Compared with those of the clonal *L*. *chinensis* from the NG area, the morphological parameters of the clonal *L*. *chinensis* from the GR area were significantly altered (Fig. 1). Approximately 15 million pairs of total clean reads were obtained in each of the GR and NG groups of clonal *L*. *chinensis*. The sequence reads generated in the present study were deposited in the NCBI Sequence Read Archive database under accession number SRP136857. In total, using the Trinity assembly software (version 2.0.2), we obtained 18,720,978 contigs, of which 91,671 had a length greater than ≥ 300 bp (Table 1). The majority of the contigs had a length in the range of 0–300 bp (Supplementary Fig. S1). The above total contigs were ultimately further assembled into 116,356 unigenes with a mean length of 668 bp and an N50 value of 1,115 bp (Table 1).

**Table 1:** Summary of the assembled data.

Item	Contigs	Unigenes
Total number	18,720,978	116,356
Total length (bp)	785,315,146	77,713,202
Mean length (bp)	42	668
N50	43	1,115

### Functional annotation and descriptive profile

Based on the results of the BLASTx analysis, the unigenes were aligned with sequences recorded in the major databases, including the Nr, Swiss-Port, GO, COG and KEGG databases (Table 2). At a cut-off *E* value of 10^−5^, 55,541 unigenes (47.73% of the total) had significant hits in the databases as follows: 55,132 in the Nr (47.38%), 32,630 in the Swiss-Prot (28.04%), 33,080 in the GO (28.43%), 10,789 in the COG (9.27%) and 6,541 in the KEGG databases (5.62%).

**Table 2:** Summary of the functional annotations of the assembled unigenes.

Public protein database	Number of unigene hits	Percentage (%)^a^
NR	55,132	47.38%
Swiss-Prot	32,630	28.04%
GO	33,080	28.43%
COG	10,789	9.27%
KEGG	6,541	5.62%

^a^Represents the proportion of the 116,356 assembled unigenes.

**Figure 2:**
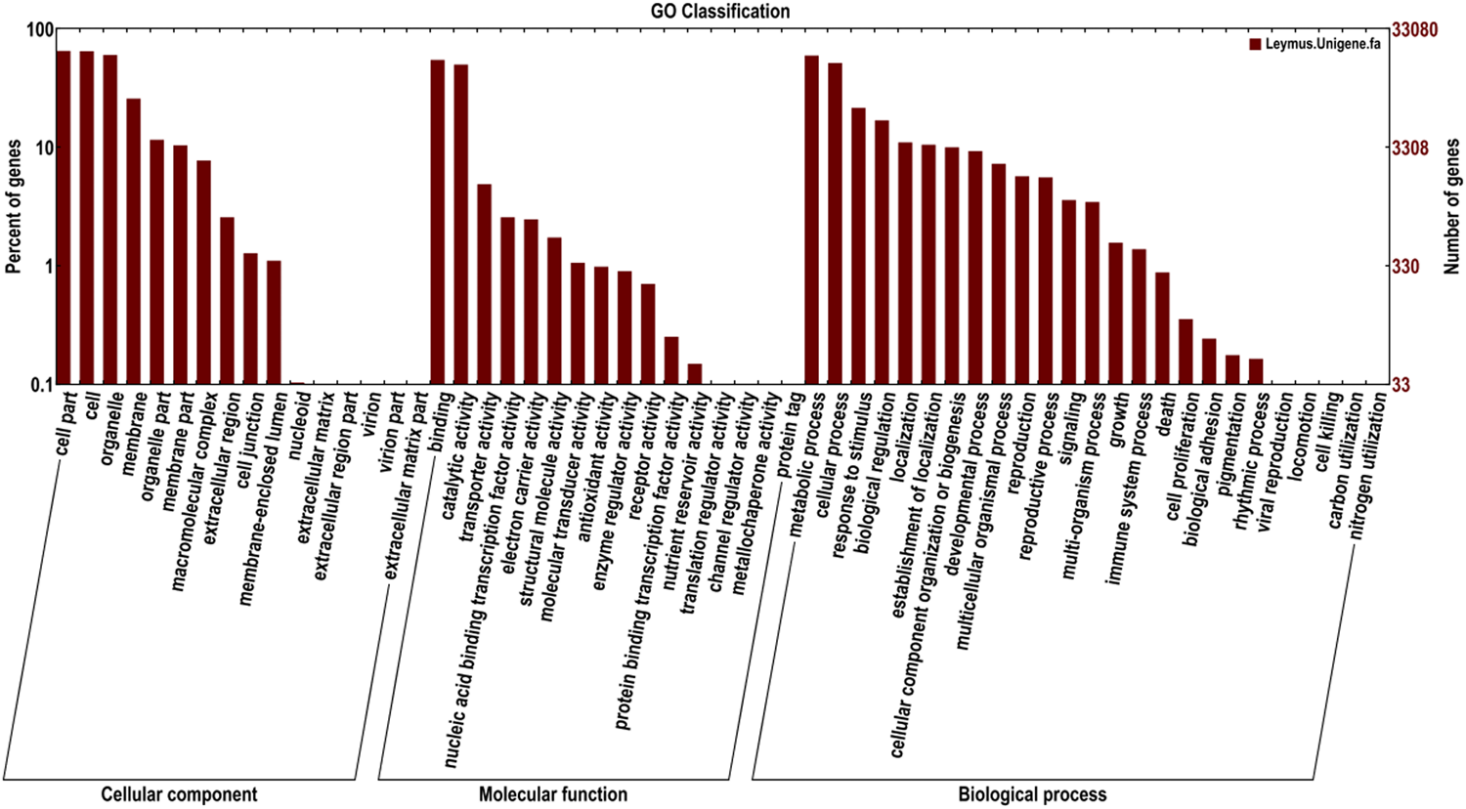
GO function classifications of unigenes for *L. chinensis*. The y-axis indicates the percentage (left) and number (right) of annotated unigenes in each term.

After the GO annotation, a total of 33,080 unigenes were assigned to one or more GO terms and were divided into 57 functional groups (Fig. 2) that belong to three categories: cellular component, molecular function and biological process. The results revealed that a high percentage of genes were assigned to “cell part”, “cell”, “organelle”, “binding”, “catalytic activity”, “metabolic process”, “cellular process” and “response to stimulus”. However, few genes were clustered as “translation regulator activity”, “channel regulator activity”, “metallochaperone activity”, “protein tag”, “nucleoid”, “extracellular matrix”, “extracellular region part”, “extracellular matrix part”, “virion”, “virion part”, “viral reproduction”, “locomotion”, “cell killing”, “carbon utilization” and “nitrogen utilization”.

**Figure 3:**
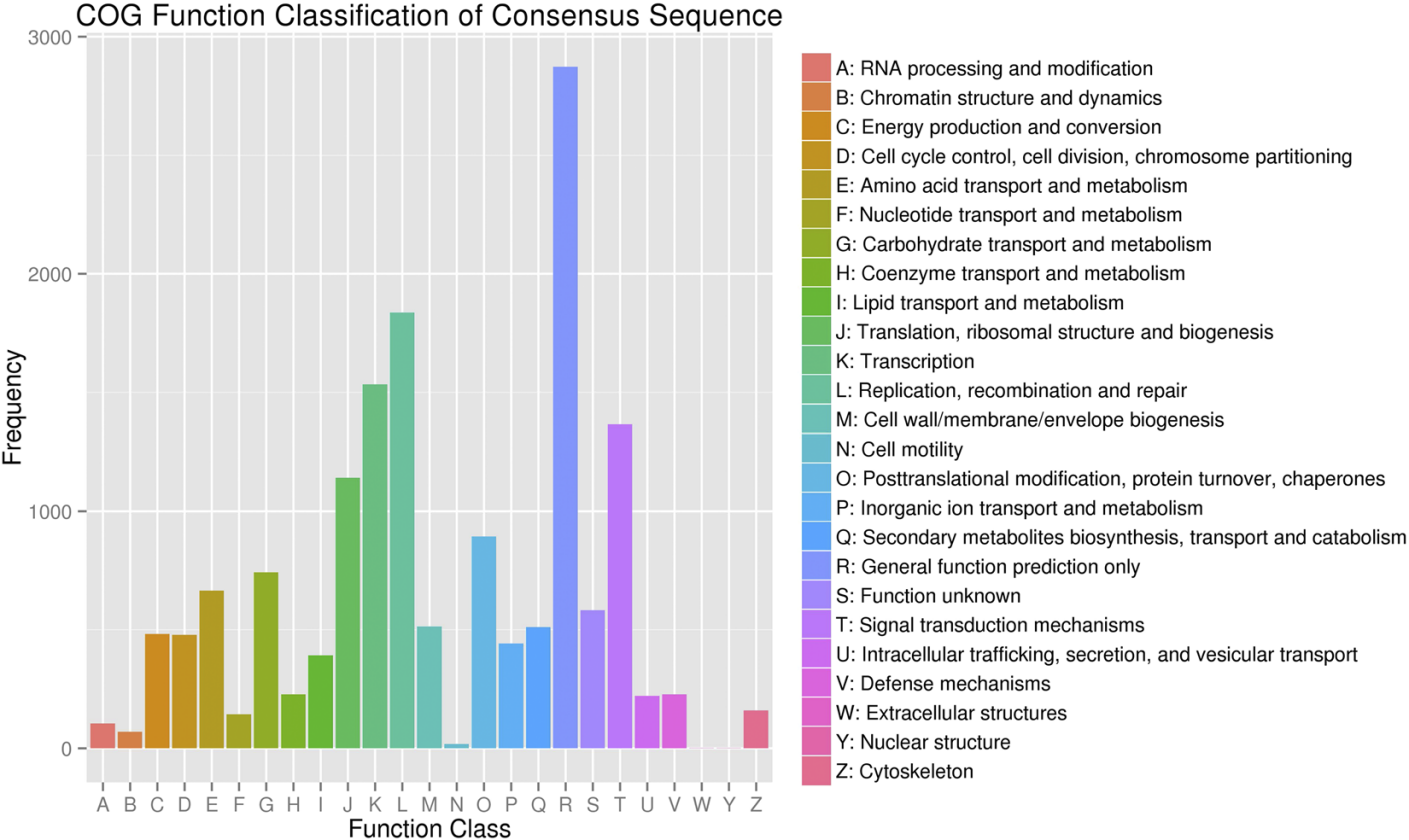
COG function classification of unigenes for *L. chinensis*. The left y-axis indicates the number of unigenes; the letters on the x-axis represent different COG categories.

**Figure 4:**
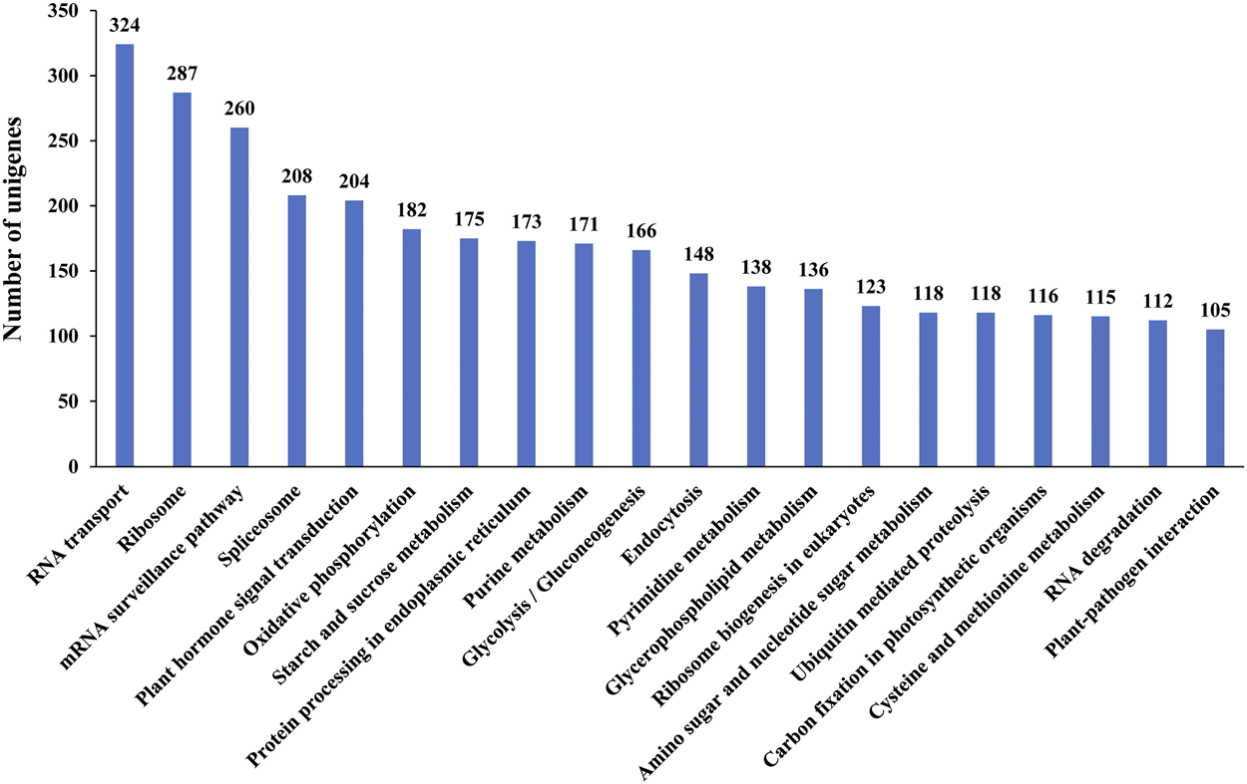
KEGG function classification for *L. chinensis*. The y-axis indicates the number of annotated unigenes; the x-axis indicates the top 20 pathways with the largest numbers of unigenes listed.

To further assess the integrity of the transcriptomic library and the effectiveness of the annotation process in this study, COG was used to classify the unigenes. In total, 10,789 unigenes were divided into 25 COG categories (Fig. 3), of which the largest group was the cluster “general function prediction” (2,873), followed by “replication, recombination and repair” (1,836); “transcription” (1,533); “signal transduction mechanisms” (1,365); and “translation, ribosomal structure and biogenesis” (1,141). The clusters “extracellular structures” (2), “nuclear structure” (2) and “cell motility” (18) represented the smallest groups. In an attempt to investigate their biological functions, we mapped the unigenes to reference canonical pathways in the KEGG databases. The results showed that 6,541 unigenes were KEGG annotated and assigned to 118 pathways. The top 20 ranked pathways containing the largest numbers of unigenes are listed in Fig. 4.

### Differentially expressed genes (DEGs) and quantitative real-time PCR (qRT-PCR) validation of clonal GR and NG *L*. *chinensis*

**Figure 5:**
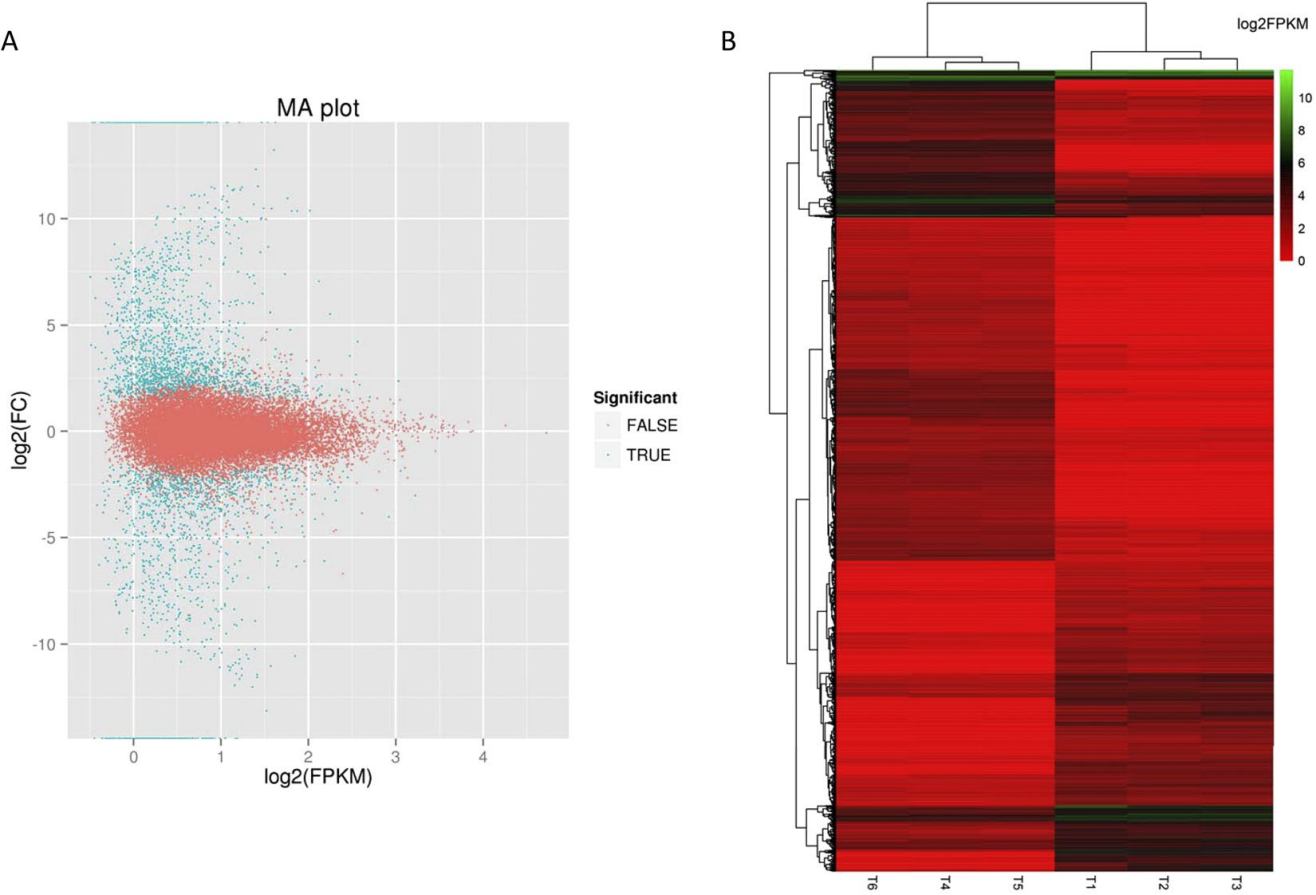
(**A**) The expression changes of DEGs in MA plots. The MA plot uses M as the y-axis (log2 fold-change) and A as the x-axis (log2 FPKM). The blue dots indicate all the DEGs, and the orange dots indicate genes without a significant difference. (**B**) Hierarchical cluster analysis of DEGs. NG group: T1, T2 and T3. GR group: T4, T5 and T6. Cluster analysis was constructed based on the log2 value of the fragments per kilobase per million reads (FPKM) of the unigenes. Green and red represent high and low expression levels, respectively.

To evaluate the differential expression profiles between clonal GR and NG *L*. *chinensis*, the potential DEGs were analysed. The fragments per kilobase per million fragments (FPKM) method was used to applied to calculate the expression levels. A false discovery rate (FDR) threshold ≤ 0.001 and the absolute value of log_2_ ratio ≥ 1 were set to obtain the DEGs. The expression levels of 3,341 unigenes significantly differed between clonal GR and NG *L*. *chinensis*; 2,024 unigenes were up-regulated, and 1,317 unigenes were down-regulated (Fig. 5A). Hierarchical clusters of the clonal GR and NG groups with these 3,341 unigenes are shown in Fig. 5B. The top 20 up-regulated and down-regulated genes are listed in Supplementary Tables S1 and S2.

Based on the Nr annotation, a total of 1,129 DEGs were assigned to one or more GO terms and classified into 57 functional categories that belong to three main categories: cellular component, molecular function and biological process (Supplementary Fig. S2). In addition, a GO functional-enrichment analysis was performed to determine the over-represented biological events and to provide a primary overview of the transcriptome of clonal *L*. *chinensis* influenced by maternal plants grown under overgrazing conditions. The results showed that two, three and seven GO terms were enriched in the cellular component, biological process and molecular function categories, respectively (Table 3).

**Table 3:** GO enrichment analysis of DEGs from GR and NG clonal *L*. *chinensis*.

GO second-level groups			
Categories	Term	Counts	*P* value
Cellular component	cytoplasmic part	27 (496)	0.00133
Cellular component	intracellular membrane-bounded organelle	28 (496)	0.0183
Biological process	response to heat	27 (729)	2.68E-06
Biological process	response to hydrogen peroxide	17 (729)	2.97E-04
Biological process	defence response by callose deposition in cell wall	7 (729)	0.0114
Biological process	response to high light intensity	15 (729)	0.0436
Molecular function	glucose-1-phosphate guanylyltransferase activity	4 (903)	0.00258
Molecular function	mannose-1-phosphate guanylyltransferase activity	4 (903)	0.00258
Molecular function	myoinositol-1-phosphate guanylyltransferase activity	4 (903)	0.00258
Molecular function	GDP-D-glucose phosphorylase activity	4 (903)	0.00258
Molecular function	galactose-1-phosphate guanylyltransferase (GDP) activity	4 (903)	0.00258
Molecular function	O-acyltransferase activity	5 (903)	0.0286
Molecular function	binding	60 (903)	0.0496

DEGs, differentially expressed genes; GR, overgrazed; NG, non-grazed.

**Figure 6:**
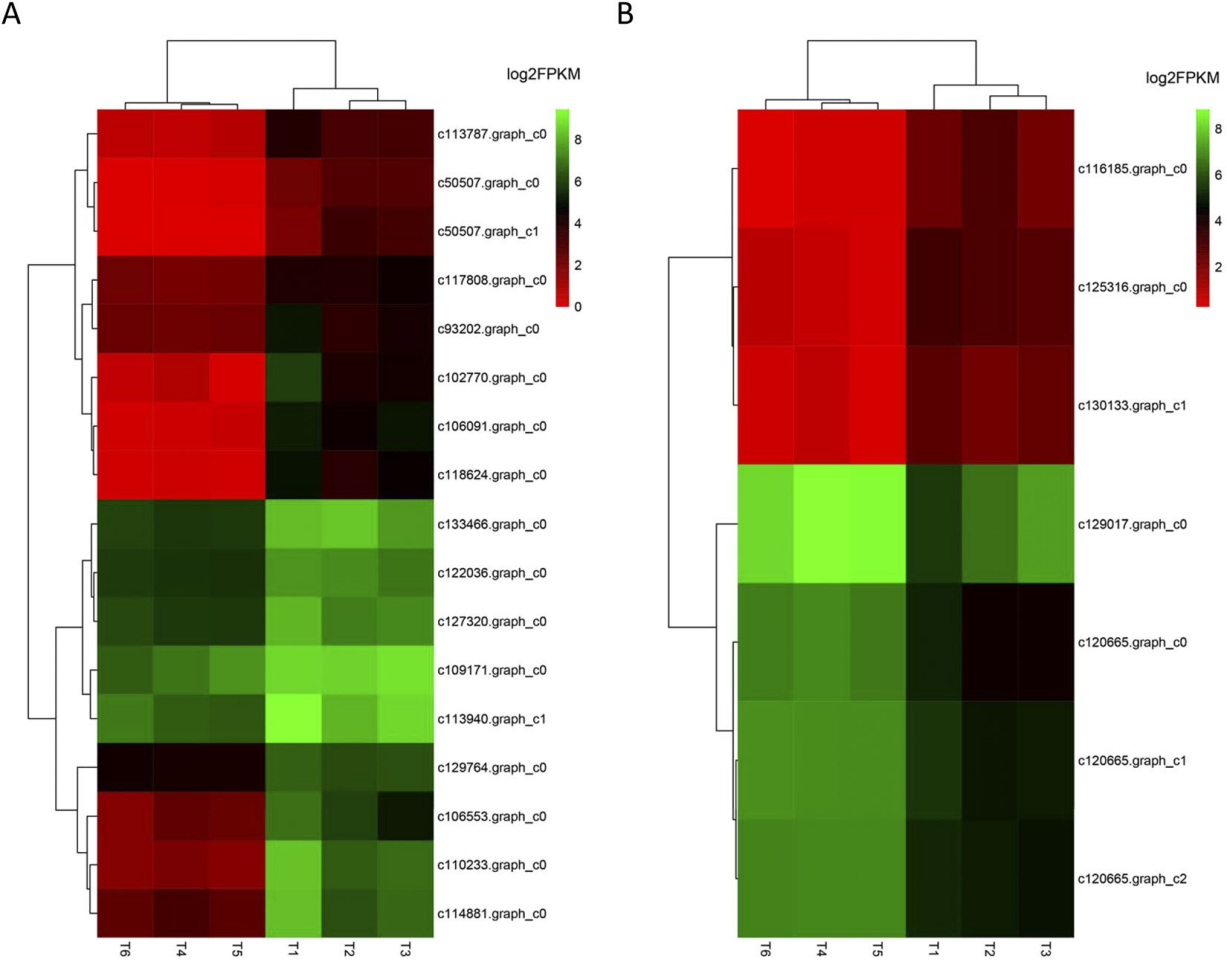
Hierarchical cluster analysis of DEGs involved in the growth and immune response of clonal *L. chinensis*. (**A**) Cluster of DEGs in the GO term “response to hydrogen peroxide”. (**B**) Cluster of DEGs in the GO term “defence response by callose deposition in cell wall”. NG group: T1, T2 and T3. GR group: T4, T5 and T6. Cluster analysis was constructed based on the log2 value of the fragments per kilobase per million reads (FPKM) of the unigenes. Green and red represent high and low expression levels, respectively.

Among the enriched GO terms, “response to hydrogen peroxide” and “defence response by callose deposition in cell wall” in the biological process category are closely related to the growth and immune response of plants. All 9 heat shock protein (HSP) genes (c110233.graph_c0, c93202.graph_c0, c114881.graph_c0, c106553.graph_c0, c113940.graph_c1, c118624.graph_c0, c102770.graph_c0, c106091.graph_c0 and c127320.graph_c0) were down-regulated in the GR group (Fig. 6A). Four GDP-L-galactose phosphorylases (GGP) genes (c129017.graph_c0, c120665.graph_c2, c120665.graph_c1 and c120665.graph_c0) were up-regulated in the GR group (Fig. 6B). As shown in Table 3, most of these genes exhibited different modes of expression in the leaf transcriptome of clonal GR *L*. *chinensis*, which may explain the dwarf-type morphological characteristics.

**Figure 7:**
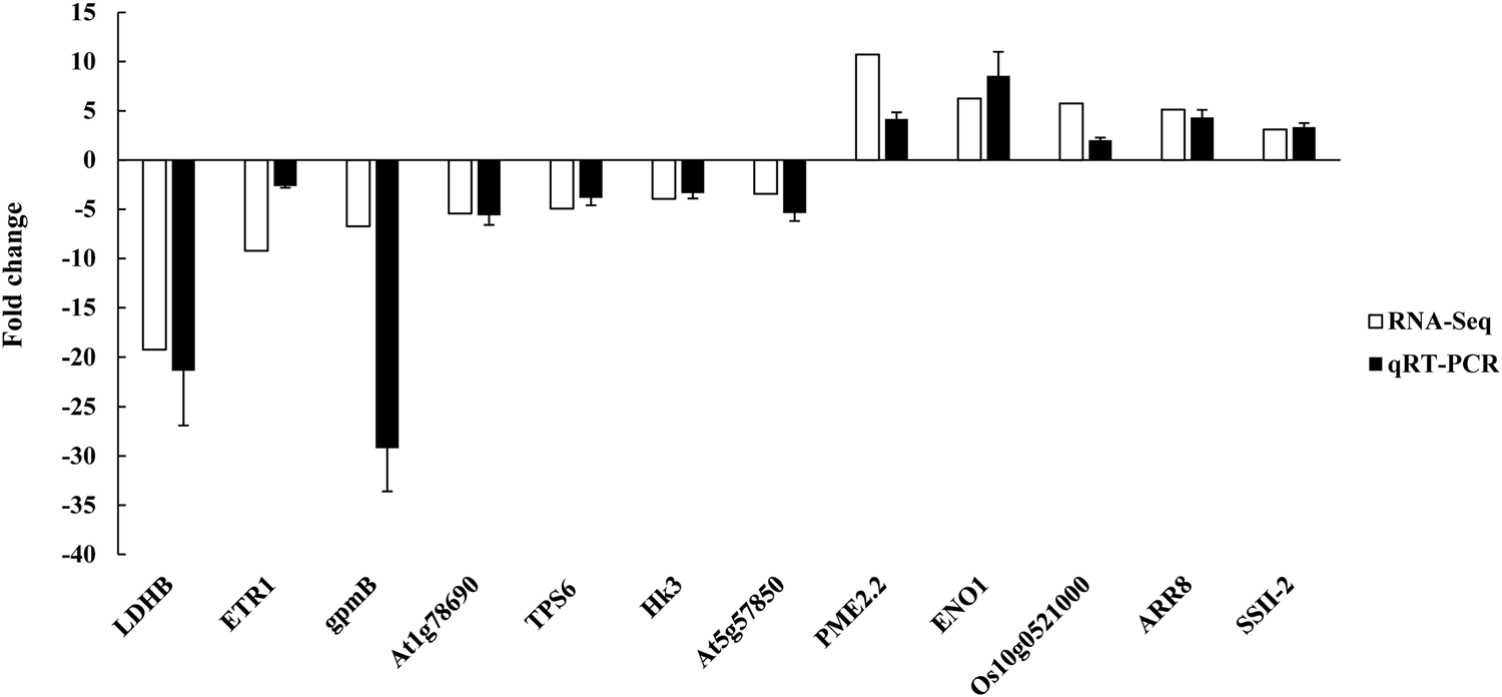
qRT-PCR validation of differentially expressed genes. The white bars represent the changes in transcript abundance determined by the FPKM method. The black bars represent the relative expression levels estimated using qRT-PCR and the 2^−ΔΔCT^ method. β-actin was used as an internal control, and the error bars indicate the standard deviation (n = 3 per group).

To validate the expression levels in the transcriptome, 12 genes whose expression patters significantly differed (5 up-regulated and 7 down-regulated) were randomly selected and tested using qRT-PCR. As a result, the relative expression levels of the 12 unigenes measured via qRT-PCR were consistent with the transcriptomic profiles obtained in the present study (Fig. 7), which suggested that our transcriptomic data were reliable.

### Measurement of oxidative-stress-related factors in clonal GR and NG *L. chinensis*

**Figure 8:**
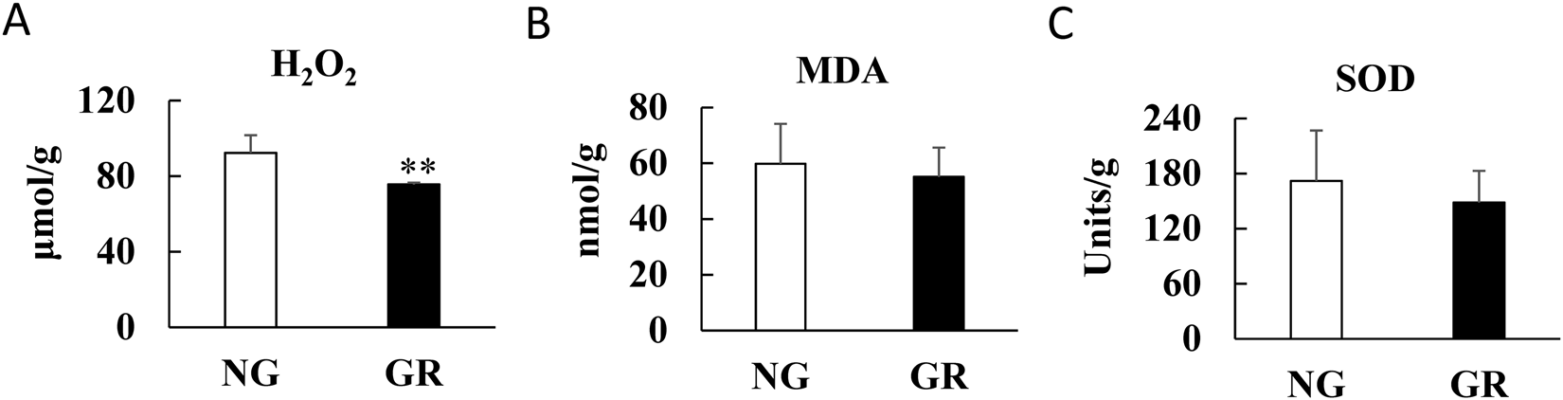
Concentrations analysis of H_2_O_2_, MDA and SOD in leaves of clonal *L. chinensis*. (**A**–**C**) H_2_O_2_, MDA and SOD. NG, non-grazed; Symbols: ^**^P < 0.01.

To confirm the physiological effect of hydrogen peroxide (H_2_O_2_) on clonal GR and NG *L. chinensis*, the concentrations of H_2_O_2_, malondialdehyde (MDA) and superoxide dismutase (SOD) were analysed using commercial kits. As shown in Fig. 8, the H_2_O_2_ level decreased significantly in the clonal GR group compared with the clonal NG group (*P* < 0.01). Interestingly, the MDA and SOD levels in the clonal GR group did not differ significantly from those in the clonal NG group. Thus, the overgrazing-induced transgenerational effects in the clonal offspring may be due to other alterations of physiological functions rather than oxidative stress.

## Discussion

Overgrazing is a frequent stress for pasture and prairie plants and is also the primary factor responsible for the current deterioration on the Inner Mongolian steppe^24^. *L*. *chinensis* is a dominant steppe species in Inner Mongolia and is well known for its tolerance to grazing and its desirable forage quality^25^. Under overgrazing conditions, *L*. *chinensis* plants growing on the steppe have developed a dwarfing tendency in recent decades. A previous study reported that the clonal offspring of white clover, a typical grassland plant, exhibited transgenerational effects after the maternal plants had experienced certain environmental stresses^26^. However, little is known about the effects of overgrazing on transcriptional activity in grassland plants. In our study, we used Illumina RNA-Seq technology to sequence six libraries prepared from samples of GR and NG groups and obtained high-quality *de novo*-assembled data. Additional comprehensive analyses provided clear evidence that overgrazing of *L*. *chinensis* plants can trigger transgenerational effects in the clonal offspring; these analyses included the annotation of *L*. *chinensis* unigenes and the evaluation of expression patterns of the *L*. *chinensis* clonal offspring collected from both GR and NG areas.

In the present study, compared with those of the NG group, the clonal offspring of *L*. *chinensis* in the GR group showed significant dwarf-type morphological characteristics, especially with respect plant development and growth. This phenomenon is consistent with the inhibition of growth due to environmental stress-induced transgenerational effects in other clonal offspring plants^26^. Our GO term enrichment analysis (Table 3) revealed that “response to hydrogen peroxide” and “defence response by callose deposition in cell wall” were significantly enriched in the GR group.

Previous research has demonstrated that the enhanced induction of genes related to defence and resistance to biotrophic pathogens was evident in transgenerational priming^27^. Long-term animal grazing is more similar to a biotic stress caused by various microorganisms in deposited animal saliva, faeces and urine, which can increase the degree of potential infection due to pathogen invasion, especially in open wounds of damaged grassland plant tissue caused by mechanical wounding^28–31^. As shown in Table 3, the DEGs associated with the GO term “defence response by callose deposition in cell wall” were found to be enriched. Of these genes, GDP-L-galactose phosphorylase (GGP) codes for an enzyme that catalyses the first key step in the synthesis of L-ascorbate and its cofactor^32^. The role of GGP in plant disease resistance has been confirmed in grapevines infected by fungi, and the expression level of the gene encoding GGP was higher and stronger in the resistant grapevine genotype than in the susceptible genotype^33^. Ethylene receptor 1 (ETR1) has a receiver domain at its C-terminus, and the binding of ethylene can induce the inactivation of ETR1^34,35^. It has been proven that the production of ethylene is steadily regulated during plant development and in response to environmental stimuli from biotic (pathogen infection) and abiotic stresses, such as hypoxia, freezing and wounding^34^. In the present study, additional defence-related genes were detected among the DEGs. Cysteine proteases are important players in the plant immune response and have been observed in fungus-infected tomatoes and bacterial pathogen-resistant Arabidopsis plants presenting an immune response^36,37^. The disease resistance protein RPM1 recognizes pathogen-encoded effector proteins, and the activation RPM1 is transduced into an efficient disease resistance response^38^. RPM1 is also up-regulated in plants under abiotic stresses such as cold acclimation^39^. The differential expression of these genes suggests that the stress memory or transgenerational effect of *L*. *chinensis* in response to long-term overgrazing can trigger defence-related gene induction in the clonal offspring. This result is consistent with the results of a transcriptomic analysis of *Stipa grandis* (Poaceae) in response to grazing^40^; the authors reported that the expression levels of most defence response-related, immune response-related and pathogenic resistant-related genes significantly differed.

Plants grown under overgrazing conditions are more likely to exhibit dwarf-type morphological characteristics^41^. The enriched GO term “response to hydrogen peroxide”, which is considered to be involved in the regulation of plant growth and development, is shown in Table 3. As a signalling molecule, hydrogen peroxide (H_2_O_2_) can be viewed as a double-edged sword since it is involved in two completely different roles in plant physiology: it is essential to aerobic metabolism and consequently, normal growth and development, but at the same time, the accumulation of H_2_O_2_, which is related to virtually all environmental stresses, is potentially damaging, as it can disrupt normal cellular processes and can even cause cell death^42,43^. Previous research has proven that the formation of H_2_O_2_ is promoted during leaf initiation and cell wall expansion^44,45^. Interestingly, in the present study, the concentration of H_2_O_2_ decreased significantly in the clonal GR group compared with the clonal NG group. Furthermore, the MDA and SOD levels were similar between the two groups. The above results indicated that H_2_O_2_ may have affected cell growth and development instead of triggering oxidative stress in the present study. Moreover, all DEGs enriched in the above GO term were down-regulated. HSPs are important molecular chaperones that play vital roles in the stabilization of proteins and assist protein refolding under stress conditions^46^. HSPs are also essential components that contribute to cellular homeostasis and are involved in protein folding, translation, assembly and degradation during ordinary cell growth and development under optimal conditions^47^. Mitogen-activated protein kinase kinase 1 and 2 (MEK) proteins are key components of mitogen-activated protein kinase (MAPK) cascades, which play critical functions in both cell proliferation and cell differentiation – the two key processes in plant growth and development^48^. Multiprotein-bridging factor 1 (MBF1) is a transcriptional co-activator that enhances the transcription of its target genes^49^. Compared with that in wild-type Arabidopsis, the suppressed function of MBF1 in transgenic Arabidopsis induced an extremely small leaf phenotype that presented much smaller leaf cells^49^. Formin-like protein (AtFH8) is required by the actin cytoskeleton of plant cells, which plays a major role in cell development^50,51^. In the present study, additional growth- or development-related genes were also observed among the DEGs. As a major group of wall glycoproteins, hydroxyproline-rich glycoproteins (HRGPs) are important polysaccharide components of cell walls and participate in plant growth and development^52^. Papain-like cysteine proteases (PLCPs) constitute a large class of proteolytic enzymes associated with plant development, the immune response, and senescence^53^. Some PLCPs carry a C-terminal granulin-like domain that functions as a plant growth hormone^54^. In the present study, the down-regulation of these DEGs in combination with the alteration of H_2_O_2_ level indicated that the dwarf-type morphological characteristics of the *L*. *chinensis* clonal offspring were closely related to the impaired functions in growth and development. Interestingly, the up-regulated DEGs were not related to oxidative stress in the *L*. *chinensis* clonal offspring. A previous study reported that *L*. *chinensis* under direct grazing stress exhibited cellular oxidative changes^55^. This finding suggests that the stress memory or transgenerational effects of overgrazing may differ for other stress mechanisms. Therefore, additional studies are needed to elucidate these mechanisms.

The present transcriptomic sequencing results greatly help elucidate the molecular-level changes in the clonal offspring of *L*. *chinensis* in response to stress memory or transgenerational effects induced by long-term overgrazing. Even when the stress of overgrazing was absent during the growth process, the *L*. *chinensis* clonal offspring still developed dwarf-type morphological characteristics, which is probably a result of the inhibited expression of genes related to growth and development. Moreover, the stress memory caused by overgrazing enhanced the expression of defence-related genes in the clonal offspring of *L*. *chinensis*, improving tolerance against stress. Overall, the results of our study provide important information on how overgrazing can impact steppe plants in the long term.

## Materials and Methods

### Ethics statement

All procedures were evaluated and approved by the Institute of Grassland Research, Chinese Academy of Agricultural Sciences (CAAS). No specific permissions were required for the *L*. *chinensis* species, and there was no involvement of any endangered or protected species.

### Field site and plant collections

The study site is located in the Xilin River catchment (43°35′N, 116°44′E), China, at an altitude of 1,214 m above sea level. The semiarid continental climate of the region featurs a mean annual temperature of 2.65 °C and a mean annual (1982–2010) precipitation of 278 mm. Generally, the highest precipitation is consistent with the maximum temperature in June, July, and August of each year. For perennial steppe plants, the growing season starts from April/May and persists to September/October, lasting for approximately 150 days. *L*. *chinensis* is one of the dominant perennial plants in typical steppe grasslands. In the present study, *L*. *chinensis* was selected as a model species for studying the relative transcriptional changes induced by transgenerational effects in response to long-term overgrazing. Samples were collected from two different plots at the experimental site: a long-term freely grazing (GR) plot and a grazing-exclusion (NG) plot. The NG plot has been enclosed since 1983 by the Institute of Grassland Research of CAAS for ecological observation and research. The GR plot (~200 ha) was located adjacent to the NG plot and has been grazed by ~600 sheep and goats annually over the last three decades. Three subplots were established within each experimental plot (GR and NG), and five 1 m × 1 m quadrats were randomly selected in each subplot. In the GR plots, temporary movable closed cages were placed in each quadrat prior to grazing before the growing season in early April 2013.

### Hydroponic experiment and sample collection

Asexual propagation in a hydroponic experiment was applied to test the effects of long-term overgrazing and non-grazing on the *L*. *chinensis* transcriptome. At the end of the *L*. *chinensis* growing season, rhizome buds in both experimental plots (GR and NG) were collected at the same time in October 2013. The rhizomes of similar length in both groups were cultivated in the laboratory under hydroponic conditions to eliminate the impact of light, water and nutrients. Twelve rhizomes with buds (5 cm each) were transferred to the same hydroponic container in 1 × Hoagland’s nutrient solution in a growth chamber^56,57^. Three hydroponic containers of *L*. *chinensis* rhizomes were cultured in each group. All containers (20 cm length × 20 cm width × 15 cm height) were randomly placed in the growth chamber (Percival, Perry, IA, USA) that provided the following conditions: a 25/15 °C day/night temperature regime, a 16 h photoperiod and 70–80% relative humidity. Artificial light was provided by 400 W lamps composed of a mixture of high-pressure sodium and metal halide bulbs, which provided a photosynthetic photon flux density of 550 µmol photons m^−2^s^−1^. The growth environment was artificially controlled to guarantee uniformity and minimize sources of variation. During the entire hydroponic experiment, nutrient solutions were constantly aerated, and the levels were maintained daily and replaced with fresh solution every 5 days. After 50 days of growth, the morphological traits, including plant height, leaf length, leaf number, leaf width and internode length, of mature plants in all the containers were measured in accordance with standard methods using a digital caliper^58^. Three *L*. *chinensis* in each group were randomly selected, and two thirds of the aboveground portion of each plant was collected in both groups. In total, 6 samples were obtained: C1, C2 and C3 (GR group) as well as T1, T2 and T3 (NG group). All harvested leaves from each experimental group were immediately frozen in liquid nitrogen and stored at −80 °C for future transcriptomic analysis.

### RNA-Seq library preparation and Illumina sequencing

The total RNA extraction was performed using TRIzol reagent (Invitrogen, Carlsbad, CA, USA). The RNA integrity was examined using the RNA Nano 6000 Assay Kit of the Agilent Bioanalyzer 2100 system (Agilent Technologies, Santa Clara, CA, USA). Six micrograms of RNA from each sample was used for transcriptome sequencing. The poly(A) mRNA was then purified from the total RNA using poly-T oligo-attached magnetic beads. The mRNA fragmentation and subsequent RNA-Seq library conversion were carried out using an mRNA-Seq library construction kit (Illumina, San Diego, CA, USA) in accordance with the manufacturer’s instructions. All library preparations were sequenced on an Illumina HiSeq 2500 platform, and paired-end reads were generated.

### Sequenced data processing and assembly

The raw sequence reads from all samples were filtered using the FASTX toolkit (http://honnonlab.cshl.edu/fastx_toolkit/). First, a perl script was used to remove all reads containing ‘N’. Adaptor sequences were then removed by the fastx_clipper program, after which bases that had a quality < 5 were removed from the 3’ end with fastq_quality_trimmer, which required a minimum sequence length of 50 bp. The reads with at least 90% of bases > 20 were then selected using a fastq quality filter for further assembly. Clean and high-quality reads were then assembled using Trinity software (version 2.0.2) (Inchworm, Chrysalis and Butterfly)^59^. Finally, the redundancy in the contigs was removed, after which the contigs were joined to obtain unique transcript fragments (unigenes).

### Functional analysis and identification of DEGs

Functional annotation of all unigenes was conducted by homology searches against various public databases, such as the Nr protein database (http://nibi.nml.nih.gov), the Swiss-Prot protein database (http://expasy.ch/sprot), the Kyoto Encyclopedia of Genes and Genomes (KEGG) pathway database (http://www.genomo.jp/kegg) and the Cluster of Orthologous Groups (COG) database (http://nibi.nml.nih.gov/COG). The GO distribution for all of the unigenes whose expression was significantly altered in the *L*. *chinensis* transcriptome were classified using the Blast2GO program^60^.

To determine the expression levels of the unigenes, the FPKM method was used. The DEGs between the NG and GR groups were identified using the DESeq R package. Heatmaps showing hierarchical clusters were constructed using the heatmap.2 package in R software^61^. An adjusted *P*-value was obtained using the Benjamini and Hochberg correction for the FDR. Genes with an adjusted *P*-value < 0.05 and a log_2_ ratio ≥ 1 were considered significant.

### Experimental validation by qRT-PCR

qPCR was performed using a StepOnePlus Real-Time PCR System (Applied Biosystems, New York, NY, USA), as previously described^62^. Actin was used as the internal control gene, and the 2^−ΔΔCT^ method was used to verify the relative quantities of 12 DEGs in the transcriptomic profile. For each qPCR analysis, three technical replicates were performed. The primers used for qPCR are listed in Supplementary Table S3.

### Measurement of cell oxidative factor concentrations in leaves

Using the same *L. chinensis* in each group, three independent replicates were performed. Leaf samples were collected following the procedure described in a previous study. Briefly, 1 cm of the cut end of each completely expanded leaf was collected, and approximately 30 cut ends were pooled^55^. All samples were frozen in liquid nitrogen and ground to a fine powder for the next measurement. The concentrations of H_2_O_2_, MDA and SOD were measured using a corresponding commercial kit (SinoBestBio, Shanghai, China) according to the manufacturer’s instructions.

### Statistical analysis

Two-tailed Student’s *t* tests were used to evaluate differences in gene expression levels, morphological traits, and concentrations of H_2_O_2_, MDA and SOD between clonal GR and NG *L*. *chinensis* using SPSS Statistics 17.0 software (SPSS, Inc., Chicago, IL, USA). A group difference was considered significant at *P* < 0.05.

## Electronic supplementary material

Supplementary Material
