# Supplementary material for: De novo transcriptomic profiling of the clonal Leymus chinensis response to long-term overgrazing-induced memory
Source: Sci Rep. 2018 Dec 17;8:17912. doi: 10.1038/s41598-018-35605-y (PMC6297159; doi:10.1038/s41598-018-35605-y)
Supplement: Supplementary file 1 — Supplementary Material [file 41598_2018_35605_MOESM1_ESM.docx]

***De novo* transcriptomic profiling of the clonal *Leymus chinensis* response to long**-**term overgrazing**-**induced memory**

Weibo Ren^1^ Xiangyang Hou^1^, Zinian Wu^1^, Lingqi Kong^1^, Huiqin Guo^2^, Ningning Hu^3^, Dongli Wan^1^, Jize Zhang^1^

^1^Institute of Grassland Research, Chinese Academy of Agricultural Sciences, Hohhot 010010, Inner Mongolia, China. ^2^College of Life Sciences, Inner Mongolia Agricultural University, Hohhot 010019, Inner Mongolia, China. ^3^Institute of Genetics and Developmental Biology, Chinese Academy of Sciences.

Address correspondence to Dr. Jize Zhang, Institute of Grassland Research, Chinese Academy of Agricultural Sciences, No. 120, Wulanchabu East Street, Saihan District, Hohhot 010010, China. Tel: +86 471 4932290; Fax: +86 471 4932290; E-mail: jzz2006@126.com


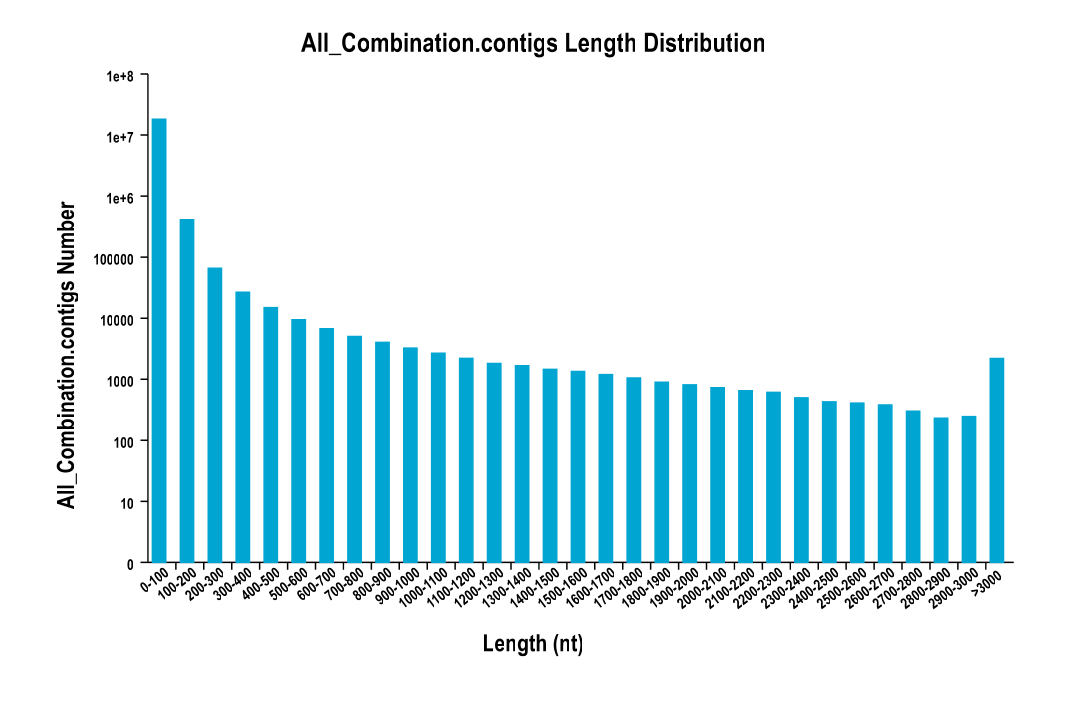


Supplementary Figure S1. Length distribution of all contigs of the *L. chinensis* transcriptome.


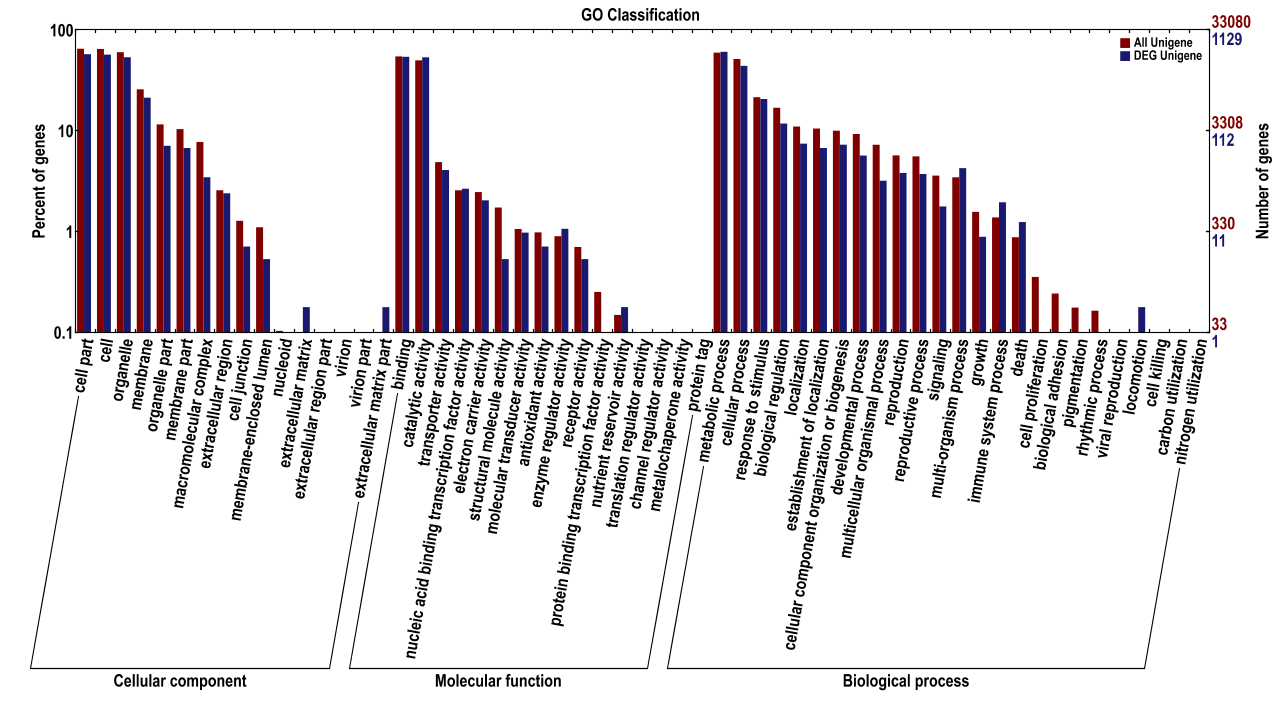


Supplementary Figure S2. Gene ontology (GO) annotation of differentially expressed genes.

Supplementary Table S1. Top 20 up-regulated genes in the GR group compared to the NG group.

| Gene ID | FDR | log2FC | Swiss-Prot annotation | Nr annotation |
| --- | --- | --- | --- | --- |
| c50167.graph_c0 | 4.17E-39 | 13.2218 | - | PREDICTED: uncharacterized protein LOC101760922 [Setaria italica] |
| c130108.graph_c0 | 4.25E-28 | 11.52469 | Putative disease resistance protein At4g19050 GN=At4g19050 OS=Arabidopsis thaliana (Mouse-ear cress) PE=3 SV=2 | Putative disease resistance RPP13-like protein 1 [Triticum urartu] |
| c89607.graph_c0 | 3.79E-23 | 11.47724 | - | PREDICTED: uncharacterized protein LOC101760504 [Setaria italica] |
| c51230.graph_c1 | 5.51E-23 | 11.46227 | Vesicle-associated protein 3-1 GN=PVA31 OS=Arabidopsis thaliana (Mouse-ear cress) PE=3 SV=1 | Cysteine-rich receptor-like protein kinase 26 [Aegilops tauschii] |
| c108694.graph_c0 | 5.76E-25 | 11.39798 | 60 kDa jasmonate-induced protein OS=Hordeum vulgare (Barley) PE=2 SV=1 | Ribosome inactivating protein, putative [Oryza sativa Japonica Group] |
| c90314.graph_c0 | 2.24E-22 | 11.37204 | - | TPA: hypothetical protein ZEAMMB73_719182 [Zea mays] |
| c51345.graph_c0 | 1.68E-24 | 11.3626 | Pollen-specific leucine-rich repeat extensin-like protein 1 (Precursor) GN=PEX1 OS=Arabidopsis thaliana (Mouse-ear cress) PE=2 SV=1 | PREDICTED: uncharacterized protein LOC100264917 [Vitis vinifera] |
| c50475.graph_c0 | 2.71E-24 | 11.12819 | Albumin-2 OS=Pisum sativum (Garden pea) PE=2 SV=1 | Pierisin [Medicago truncatula] |
| c126734.graph_c1 | 7.52E-23 | 11.12754 | Proline-rich receptor-like protein kinase PERK2 GN=PERK2 OS=Arabidopsis thaliana (Mouse-ear cress) PE=2 SV=3 | hypothetical protein F775_21803 [Aegilops tauschii] |
| c103152.graph_c0 | 5.45E-24 | 11.08681 | Mitochondrial outer membrane protein porin 6 GN=VDAC6 OS=Oryza sativa subsp. japonica (Rice) PE=2 SV=1 | hypothetical protein PRUPE_ppa009817mg [Prunus persica] |
| c135169.graph_c0 | 2.45E-20 | 11.05937 | Leucine-rich repeat extensin-like protein 5 (Precursor) GN=LRX5 OS=Arabidopsis thaliana (Mouse-ear cress) PE=2 SV=2 | ZYRO0A12386p [Zygosaccharomyces rouxii] |
| c135083.graph_c0 | 1.33E-19 | 10.90439 | - | cysteine protease, putative [Ricinus communis] |
| c51596.graph_c0 | 1.30E-20 | 10.81688 | - | hypothetical protein [Oryza sativa Japonica Group] |
| c119086.graph_c0 | 2.86E-31 | 10.7567 | Disease resistance protein RPM1 GN=F17A9.20 OS=Arabidopsis thaliana (Mouse-ear cress) PE=1 SV=1 | Disease resistance protein RPM1 [Aegilops tauschii] |
| c88165.graph_c0 | 1.04E-30 | 10.67619 | - | PREDICTED: uncharacterized protein LOC100836056 [Brachypodium distachyon] |
| c135430.graph_c0 | 1.59E-19 | 10.58048 | - | hypothetical protein SORBIDRAFT_05g010901 [Sorghum bicolor] |
| c47175.graph_c0 | 3.71E-35 | 10.53332 | - | hypothetical protein F775_16657 [Aegilops tauschii] |
| c76932.graph_c0 | 2.25E-33 | 10.48659 | Thioredoxin H-type 2 GN=THL-2 OS=Brassica napus (Rape) PE=2 SV=1 | Thioredoxin H-type [Triticum urartu] |
| c135060.graph_c0 | 7.22E-26 | 10.48351 | Leucine-rich repeat extensin-like protein 5 (Precursor) GN=LRX5 OS=Arabidopsis thaliana (Mouse-ear cress) PE=2 SV=2 | hypothetical protein F775_32944 [Aegilops tauschii] |
| c76509.graph_c0 | 3.84E-33 | 10.46923 | - | TPA: hypothetical protein ZEAMMB73_719182 [Zea mays] |

Supplementary Table S2. Top 20 down-regulated genes in the GR group compared to the NG group.

| Gene ID | FDR | log2FC | Swiss-Prot annotation | Nr annotation |
| --- | --- | --- | --- | --- |
| c135080.graph_c0 | 4.76E-19 | -10.3984 | - | hypothetical protein F775_06221 [Aegilops tauschii] |
| c135184.graph_c0 | 7.29E-18 | -10.4544 | Extensin (Precursor) GN=HRGP OS=Sorghum bicolor (Sorghum) PE=3 SV=1 | hypothetical protein GUITHDRAFT_119564 [Guillardia theta CCMP2712] |
| c50154.graph_c0 | 5.94E-18 | -10.4951 | - | predicted protein [Hordeum vulgare subsp. vulgare] |
| c134890.graph_c0 | 4.17E-39 | -10.5679 | - | hypothetical protein F775_06221 [Aegilops tauschii] |
| c96326.graph_c0 | 1.55E-18 | -10.6678 | Extensin (Precursor) GN=HRGP OS=Zea mays (Maize) PE=2 SV=1 | hypothetical protein VOLCADRAFT_86751 [Volvox carteri f. nagariensis] |
| c51378.graph_c0 | 5.50E-09 | -10.6875 | 60 kDa jasmonate-induced protein OS=Hordeum vulgare (Barley) PE=2 SV=1 | predicted protein [Hordeum vulgare subsp. vulgare] |
| c50203.graph_c0 | 5.77E-10 | -10.7072 | Probable LRR receptor-like serine/threonine-protein kinase At3g47570 (Precursor) GN=At3g47570 OS=Arabidopsis thaliana (Mouse-ear cress) PE=1 SV=1 | LRR receptor-like kinase [Triticum aestivum] |
| c85305.graph_c0 | 8.68E-07 | -10.7272 | 3'-N-debenzoyl-2'-deoxytaxol N-benzoyltransferase GN=TAX10 OS=Taxus canadensis (Canadian yew) PE=1 SV=1 | 3&apos; -N-debenzoyl-2&apos; -deoxytaxol N-benzoyltransferase [Aegilops tauschii] |
| c73325.graph_c0 | 1.63E-07 | -10.8323 | Putative ribonuclease H protein At1g65750 GN=At1g65750 OS=Arabidopsis thaliana (Mouse-ear cress) PE=3 SV=1 | PREDICTED: uncharacterized protein LOC100845261 [Brachypodium distachyon] |
| c110691.graph_c0 | 1.32E-11 | -10.871 | - | hypothetical protein F775_13915 [Aegilops tauschii] |
| c51531.graph_c0 | 2.25E-33 | -10.9136 | Cytosolic sulfotransferase 8 GN=T6J4.16 OS=Arabidopsis thaliana (Mouse-ear cress) PE=2 SV=1 | Flavonol sulfotransferase-like protein [Triticum urartu] |
| c125295.graph_c0 | 9.83E-05 | -10.9924 | Ananain (Precursor) GN=AN1 OS=Ananas comosus (Pineapple) PE=1 SV=2 | papain-like cysteine proteinase [Hordeum vulgare subsp. vulgare] |
| c46217.graph_c0 | 5.57E-07 | -11.0341 | - | PREDICTED: lipoxygenase homology domain-containing protein 1-like [Setaria italica] |
| c110677.graph_c0 | 3.35E-20 | -11.0422 | - | hypothetical protein F775_01963 [Aegilops tauschii] |
| c135072.graph_c0 | 8.17E-23 | -11.0877 | - | salt-induced protein [Leymus chinensis] |
| c134958.graph_c0 | 4.29E-06 | -11.1506 | Probable carboxylesterase 18 GN=CXE18 OS=Arabidopsis thaliana (Mouse-ear cress) PE=2 SV=1 | predicted protein [Hordeum vulgare subsp. vulgare] |
| c122235.graph_c1 | 4.73E-20 | -11.3913 | Formin-like protein 13 GN=FH13 OS=Arabidopsis thaliana (Mouse-ear cress) PE=2 SV=3 | hypothetical protein SELMODRAFT_119308 [Selaginella moellendorffii] |
| c51181.graph_c1 | 1.64E-09 | -11.9177 | Aspartic proteinase nepenthesin-1 (Precursor) GN=nep1 OS=Nepenthes gracilis (Slender pitcher plant) PE=1 SV=1 | TPA: hypothetical protein ZEAMMB73_214154 [Zea mays] |
| c51231.graph_c0 | 6.20E-07 | -11.9983 | Horcolin OS=Hordeum vulgare (Barley) PE=3 SV=1 | OSJNBa0016N04.16 [Oryza sativa Japonica Group] |
| c50203.graph_c1 | 7.29E-10 | -13.1275 | Putative receptor-like protein kinase At3g47110 (Precursor) GN=At3g47110 OS=Arabidopsis thaliana (Mouse-ear cress) PE=3 SV=1 | PREDICTED: probable LRR receptor-like serine/threonine-protein kinase At3g47570-like [Brachypodium distachyon] |

Supplementary Tables S3. The qPCR primers used for verification of the differentially expressed genes.

| Gene name | Primer sequence (5'→3') |
| --- | --- |
| β-actin | forward: GCACCCTGTGTTGCTCACT  reverse: TACCTTGATCTTCATGCTGCTC |
| LDHB | forward: CCTGACCACAAGAACTCTGAC  reverse: CGGCACAGTCTTCTAGTACAG |
| ETR1 | forward: ACCATATTTTAAGTATGAGCCTAGAC  reverse: TTAAAACTGACAAAATTCAGAAG |
| gpmB | forward: TGTTCAGGTACGAGACGCA  reverse: CTGTTCTGTTCTGCCACTTCTC |
| At1g78690 | forward: GCACATAGCGTCCATCTCAG  reverse: CAAGCCTCTTCTAACGGTCAG |
| TPS6 | forward: CAGCCAGATTACCAGCCAG  reverse: ATAAGATTCATGCCATCCCTCAC |
| Hk3 | forward: GATGCTCCTCACCTTCGTC  reverse: ACTCTAAAGTCCGTTCCTCCA |
| At5g57850 | forward: CGCTTCGACAAGATCCTGAG  reverse: CTGAGCCTTCCATCCTCCA |
| PME2.2 | forward: TCTAATCCGTTACACAACAATCGCC  reverse: TGACCAAGAGAACTTTGCTAGGG |
| ENO1 | forward: CCAGTCAGCCAACAAGCAG  reverse: GCATCCACCAACGAAGTAGAG |
| Os10g0521000 | forward: CAGGAATAGCAACTTCAGTATCCA  reverse: AACCAGAGTTCAGCAATCCC |
| ARR8 | forward: AAGTTACCACCGTGGATTCCGGGA  reverse: AGGCATGCAGTAGTCAGTGATG |
| SSII-2 | forward: CTTTCCAGTAACCCGATGCC  reverse: ATCCGTCAGCTCCTGTCTC |
